# Association between medication adherence and blood pressure control and factors associated with antihypertensive medication adherence in the Melaka Tengah District: A cross-sectional survey

**DOI:** 10.51866/oa.651

**Published:** 2024-10-17

**Authors:** Siti Zaleha Suleiman, Mila Nu Nu Htay, Htoo Htoo Kyaw Soe, Li Ying Cherlynn Low, Siti Hawa Alias, Syamimi Yussof, Wei Xiong Keng, Karleen Chong, Mohammad Faiz Sahiran, Muhamad Hafiz Harun, Azman Othman, Rosmiza Abdullah, Nadratulmazlyna Mohd Mansor, Nor Haslinda Ishak, Ahmad Fithri Azam Abdul Rahman, Soe Moe

**Affiliations:** 1 MBBS, DTM&H, EDEM, MTID, MFM, PhD, Department of Community Medicine, Faculty of Medicine, Manipal University College Malaysia (MUCM), Jalan Batu Hampar, Bukit Baru, Melaka, Malaysia. Email: drmlnnh@gmail.com; 2 MD, MFamMed, Klinik Kesihatan Ayer Keroh, Jalan Ayer Keroh Lama, Melaka, Malaysia.; 3 MBBS, MPH, PhD, Department of Community Medicine, Faculty of Medicine, Manipal University College, Jalan Batu Hampar, Bukit Baru, Melaka, Malaysia.; 4 MBBS, MAFP, FRACGP, Klinik Kesihatan Bukit Rambai, Jalan Bukit Rambai, Bukit Rambai, Melaka, Malaysia.; 5 MBBS, MAFP, FRACGP, Klinik Kesihatan Cheng, Taman Cheng Perdana, Melaka, Malaysia.; 6 MBBS, MAFP, FRACGP, Klinik Kesihatan Peringgit, Jalan Pantai Peringgit, Melaka, Malaysia.; 7 MD, MFamMed, Klinik Kesihatan Ayer Keroh, Jalan Ayer Keroh Lama, Melaka, Malaysia.; 8 MD, MFamMed, Klinik Kesihatan Tengkera, Jalan Tengkera, Kampung Lapan, Melaka, Malaysia.; 9 MBBS, MFamMed, Klinik Kesihatan Seri Tanjung, Tanjung Kling Melaka Tengah, Melaka, Malaysia.; 10 MBBS, MFamMed, Klinik Kesihatan Peringgit, Jalan Pantai Peringgit, Melaka, Malaysia.; 11 MBBS, MFamMed, Klinik Kesihatan Peringgit, Jalan Pantai Peringgit, Melaka, Malaysia.; 12 MBBCh.BAO.LRCP & SI, MFamMed, Klinik Kesihatan Batu Berendam, Jalan Tunku Abdul Rahman, Kampung Sungai Putat, Melaka, Malaysia.; 13 MBBS, MFamMed, Klinik Kesihatan Ayer Molek, Jalan Ayer Molek / Jasin, Kampung Permatang, Melaka, Malaysia.; 14 MD, MFamMed, Klinik Kesihatan Batu Berendam, Jalan Tunku Abdul Rahman, Kampung Sungai Putat, Melaka, Malaysia.; 15 MD, MFamMed, Klinik Kesihatan Tengkera, Jalan Tengkera, Kampung Lapan, Melaka, Malaysia.; 16 MBBS, MMedSc, M.A, MRes, Department of Community Medicine, Faculty of Medicine, Manipal University College, Jalan Batu Hampar, Bukit Baru, Melaka, Malaysia.

**Keywords:** Hypertension, Drug therapy, Primary health care, Malaysia

## Abstract

**Introduction:**

The burden of cardiovascular disease and early morbidity and mortality is exacerbated by hypertension. According to the 2019 National Health and Morbidity Survey, 30% of adults in Malaysia aged over 18 years had hypertension. Our study aimed to investigate the association between medication adherence and blood pressure control and the factors associated with antihypertensive medication adherence.

**Methods:**

This cross-sectional study was conducted among adult patients with hypertension who had been under treatment at public primary health clinics in the Melaka Tengah District. A self-administered questionnaire was used, and blood pressure was measured. Descriptive and multivariate logistic regression analyses were conducted using the Statistical Package for the Social Sciences (version 28).

**Results:**

A total of 1531 patients were recruited in this study. Among them, 74.1% had good antihypertensive medication adherence, and 51.4% had their blood pressure controlled. Medication adherence was significantly associated with blood pressure control (P<0.005). The multivariate analysis showed that the determinants for antihypertensive medication non-adherence were Malay ethnicity, secondary education, farther distance from the clinic, experience of side effects of antihypertensive medications, concern about long-term side effects and usage of alternative medicine (P<0.05). Taking multiple antihypertensive medications was the only factor associated with uncontrolled blood pressure (P<0.05).

**Conclusion:**

Addressing issues on medication adherence is important to ensure blood pressure control. The factors associated with non-adherence should be closely monitored to improve blood pressure control and prevent adverse health outcomes. Single-pill combination antihypertensive medications are encouraged to reduce pill burden and improve blood pressure control.

## Introduction

The burden of cardiovascular disease and early morbidity and mortality is exacerbated by hypertension.^1^ In 2019, over 34% of men and 32% of women had hypertension based on a report by the Non-Communicable Diseases Risk Factor Collaboration,^2^ and less than 21% of individuals with hypertension had their blood pressure under control according to the World Health Organization.^3^ The 2019 National Health and Morbidity Survey (NHMS) showed that 30% of adults in Malaysia aged over 18 years had hypertension.^4^ Previous studies have also shown that Malaysia has a greater prevalence of hypertension than its neighbouring nations, such as Singapore (21.7%)^5^ and Thailand (24.7%).^6^

The term ‘non-adherence’ is used to describe a situation wherein a patient consistently disregards treatment rather than merely temporarily forgetting to take a prescription or complete a prescribed course of therapy.^7^ Many healthcare professionals are concerned with medication non-adherence, as it results in inefficient health spending, which has an immediate impact on the provision of healthcare services and burdens the nation in the current environment of limited resources.^7^

Multiple factors have been found to be associated with adherence to antihypertensive medications across different countries. A nationwide population-based study in Korea showed that adherence consistently increased as age increased until 69 years and decreased starting from the age of 70 years.^8^ Other studies reported that residence in rural areas^9^ and ethnic variations^10^ were significantly associated with adherence to antihypertensive medications. Moreover, the number of antihypertensive drugs^11^ and the presence of side effects such as excessive urination and decreased sexual drive^12^ influenced medication adherence.

Despite the advent of technology and newer drugs in the market, the increasing prevalence of hypertension in Malaysia remains a challenge. According to the NHMS, the prevalence of hypertension in the country has been rising steadily over the last decade.^4,13^ Nonadherence has been mostly blamed for the failure of antihypertensive treatment, reducing its maximum clinical benefit.^7^ Therefore, it is imperative to evaluate the association between medication adherence and blood pressure control and the factors associated with antihypertensive medication adherence to reveal the potential modifiable barriers in managing hypertension and improve clinical care in a shorter period and lower cost.

## Methods

### Study setting and population

This cross-sectional study was conducted in Melaka, Malaysia, recruiting patients with active hypertension across all public primary health clinics under the Melaka Tengah District. The study was conducted from March to November 2023.

### Sample size and sampling

The sample size was calculated based on the study conducted by Chia et al.^14^ using the G*Power software version 3.1.9.2, with a power of 80% and a significance level (a) of 0.05. Given a nonresponse rate of 20%, 1516 participants were needed to be recruited in this study.

Patients (i) aged >18 years and under treatment for hypertension, (ii) with an active hypertension case not lasting <12 months and (iii) with concomitant dyslipidaemia were included in the study. Conversely, patients (i) with an active case who defaulted follow-up for >6 months, (ii) who were pregnant, (iii) who sought treatment in the clinic for emergency cases and (iv) who had concomitant diabetes, end-stage renal failure or other illness were excluded from this study.

### Study instrument

The study instrument assessed the following: (i) sociodemographic characteristics, (ii) medication adherence, (iii) clinical characteristics and (iv) patient-related factors leading to non-adherence.

Medication adherence was measured using the previously validated Malaysia Medication Adherence Assessment Tool (MyMAAT). The tool consists of 12 items, and the responses are scored on a 5-point Likert scale *(1=strongly agree, 5=strongly disagree*).^15^

The clinical characteristics included the duration of hypertension, duration of drug use, frequency of drug use, number of antihypertensive drugs, usage of combination drugs, total number of all medications, combination of antiplatelet agents, combination with statins and other comorbidities.

The patient-related factors included the family history of hypertension, experience of hypertensive emergency, experience of side effects of antihypertensive medications, concern about long-term effects, availability of family support and usage of traditional/alternative medicine.

### Data collection

A self-administered questionnaire was used to collect participants’ information after obtaining their informed consent. Participants’ blood pressure was measured according to the 2018 Malaysian Clinical Practice Guideline for the Management of Hypertension.^16^ Two readings were taken 1 minute apart from the same arm in the same position. The higher reading was used. A third reading was taken when the difference between the first two readings was >10 mmHg. When a third reading was required, the average between the second and third readings was used.

### Data analysis

Data were entered into Microsoft Excel and analysed using the IBM Statistical Package for the Social Sciences version 28, Armonk, NY: IBM Corp. Sociodemographic and clinical data were summarised descriptively. Categorical data were presented as frequencies and percentages and continuous data as means [standard deviations (SDs)]. The association between blood pressure control and medication adherence was investigated through multiple logistic regression, adjusting for all sociodemographic, clinical and patient-related factors.

### Ethical considerations

The study was conducted in compliance with the ethical principles outlined in the Declaration of Helsinki and the Malaysian Good Clinical Practice Guideline. Approval from the National Medical Research Register (NMRR) and Medical Research & Ethics Committee (MREC) was obtained before the initiation of the study.

Thereafter, approval was obtained from Pejabat Kesihatan Daerah Melaka Tengah.

## Results

A total of 1531 patients were recruited in the study. Among them, 55.1% were 60 years old and above, and 65.4% were women. More than half of the participants were women (60.4%) and completed secondary education (56.5%). The majority were married (82.6%) and were staying with their family members (92.0%). Approximately 41.5% of the participants were employed, and 91.7% had a monthly family income of <RM 5000 (Table 1).

**Table 1:** Sociodemographic characteristics of the participants (n=1531).

Variable	n (%)
**Age**	
<60 years	687 (44.9)
≥60 years	844 (55.1)
Mean (SD)	60(11.97)
Minimum maximum	20-94
**Sex**	
Male	668 (43.6)
Female	863 (56.4)
**Ethnicity**	
Malay	924 (60.4)
Chinese	534 (34.9)
Indian	50 (3.3)
Ochers	23(1.5)
**Educational level**	
Primary	404 (26.4)
Secondary	865 (56.5)
Tertiary	262 (17.1)
**Marital status**	
Married	1264 (82.6)
Single/divoreed/widowed	267 (17.4)
**Occupation**	
Employed	635 (41.5)
Unemployed	896 (58.5)
**Income**	
<RM 5000	1404 (91.7)
RM 5001-11,000	110(7.2)
>RM 11,000	16(1.0)

Among the participants, 87% lived within 5 km from the primary healthcare clinic. Approximately 72.4% reported that they had a family history of hypertension. The patient-related factors are detailed in Appendix Table 1.

Regarding clinical factors, the mean duration of hypertension was 8.01 years (SD=6.08), and the mean duration of antihypertensive treatment was 7.86 years (SD=6.02). Regarding antihypertensive medications, 93% of the participants were taking drugs once daily, while 53.9% were taking a single drug. The clinical factors of the participants are shown in Appendix Table 2.

**Appendix Table 1:** Patient-related factors among the participants (n=1531).

Variable	n (%)
**Staying with family members**	
Yes	1400 (91.4)
No	131 (8.6)
**Distance from the clinic**	
0-<5 km	1332 (87.0)
5-10 km	159 (10.4)
>10 km	22 (1.4)
**Family history of hypertension**	
Yes	1108 (72.4)
No	423 (27.6)
**Experience of hypertensive emergency**	
Yes	143 (9.3)
No	1388 (90.7)
**Experience of side effects of antihypertensive medications**	
Yes	132 (8.6)
No	1399 (91.4)
**Concern about the long-term side effects of medications**	
Yes	399 (26.1)
No	1132 (73.9)
**Usage of alternative medicine**	
Yes	120 (7.8)
No	1411 (92.2)

**Appendix Table 2:** Clinical factors among the participants (n=1531).

Variable	n (%)
**Duration of hypertension**	
Mean (SD)	8.01 (6.08)
Minimum-maximum	1-40
1-5 years	660 (43.1)
5-10 years	527 (34.4)
>10 years	344 (22.5)
**Duration of antihypertensive treatment**	
Mean (SD)	7.86 (6.02)
Minimum-maximum	0.5-40
1-5 years	681 (44.5)
5-10 years	520 (34.0)
>10 years	329 (21.5)
**Frequency of taking medications**	
Once daily	1424 (93.0)
Twice daily	90 (6.9)
Thrice daily	17(1.1)
**Number of antihypertensive medications**	
1	806 (53.9)
2	504 (33.7)
3	184 (12.3)
**Taking combined antihypertensive medications**	
Yes	58 (3.8)
No	1473 (96.2)
**Total number of medications taking daily (all medications taken by patient)**	
1-2	815 (53.2)
3-4	559 (36.5)
>4	157(10.3)
**Combination with antiplatelet medications**	
Yes	199(13.0)
No	1332 (87.0)
**Combination with statins**	
Yes	1029 (67.2)
No	502 (32.8)
**Having other comorbidities**	
Yes	1069 (69.8)
No	462 (30.2)

The majority of the participants (74.10%) reported to adhere to antihypertensive medications. Slightly more than half of the participants (51.40%) had their blood pressure controlled.

Table 2 presents the sociodemographic, patient-related and clinical factors associated with non-adherence to antihypertensive medications. In the multivariate logistic regression analysis, some sociodemographic factors such as ethnicity and educational level were significantly associated with non-adherence. The Malay participants were more likely to be non-adherent than the Chinese participants [adjusted odds ratio (aOR)=1.64, 95% CI=1.24, 2.17]. The participants ho completed secondary education were more likely to be non-adherent than those who received tertiary education (aOR=1.61, 95% CI=1.12, 2.31). Among the patient-related factors, the istance from the clinic, experience of side effects of antihypertensive mediations, concern about long-term side effects and usage of alternative medicine were significantly associated with nonadherence. The participants living more than 10 km from the clinic were 3.30 times more likely to be non-adherent than those living less than 5 km from the clinic (aOR=3.30, 95% CI=1.37, 7.97). The participants who had experienced side effects of antihypertensive medications were more likely to be non-adherent than those who did not (aOR=1.63, 95% CI=1.10, 2.43). Conversely, the participants who expressed concerns about the long-term side effects of antihypertensive medications were more likely to be non-adherent than those who did not (aOR=1.72, 95% CI=1.33, 2.24). The participants who used alternative medicine were also more likely to be nonadherent than those who did not (aOR=1.93, 95% CI=1.29, 2.88) (Table 2).

**Table 2:** Factors associated with non-adherence to antihypertensive medications among the participants (n=1531).

Variable	Adherence n (%)	Non-adherence n (%)	Univariate analysis		Multivariate analysis	
			OR (95% Cl)	P	aOR (95% Cl)	P
**Sociodemographic factors**						
**Age**						
≥60 years	649 (76.9)	195 (23.1)	Reference			
<60 years	485 (70.6)	202 (29.4)	1.39 (1.10, 1.74)	0.005	1.01 (0.77, 1.33)	0.950
**Sex**						
Male	495 (74.1)	173(25.9)	Reference			
Female	639 (74.0)	224 (26.0)	1.00 (0.80, 1.26)	0.980		
**Ethnicity**						
Chinese	435 (81.5)	99(18.5)	Reference			
Malay	643 (69.6)	281 (30.4)	1.92(1.48, 2.49)	<0.001	1.64 (1.24, 2.17)	<0.001
Indian	39 (78.0)	11 (22.0)	1.24 (0.61,2.51)	0.550	1.14 (0.56, 2.35)	0.721
Others	17 (73.9)	6(26.1)	1.55 (0.60,4.03)	0.368	1.40 (0.52, 3.77)	0.503
**Educational level**						
Tertiary	203 (77.5)	59 (22.5)	Reference			
Secondary	615 (71.1)	250 (28.9)	1.40(1.01, 1.94)	0.043	1.61 (1.12, 2.31)	0.010
Primary	316(78.2)	88 (21.8)	0.96 (0.66, 1.39)	0.823	1.33 (0.87, 2.04)	0.191
**Marital status**						
Single/divorced/widowed	206 (77.2)	61 (22.8)	Reference			
Married	928 (73.4)	336 (26.6)	1.22 (0.90, 1.67)	0.206	1.02 (0.73, 1.42)	0.911
**Occupation**						
Employed	465 (73.2)	170 (26.8)	Reference			
Unemployed	669 (74.7)	227 (25.3)	0.93 (0.74, 1.17)	0.527		
**Income**						
>RM 11,000	14 (87.5)	2 (12.5)	Reference			
RM 5001-11,000	77 (70.0)	33 (30.0)	3.00 (0.65, 13.95)	0.161	3.09 (0.65, 14.71)	0.157
<RM 5000	1042 (74.2)	362 (25.8)	2.43 (0.55, 10.75)	0.241	251 (0.55, 11.51)	0.236
**Patient-related factors**						
**Staying with family members**						
Yes	1041 (73.9)	367 (26.1)	Reference			
No	93 (75.6)	30 (24.4)	0.92 (0.60, 1.40)	0.684		
**Receiving family support**						
Yes	1040 (74.3)	360 (25.7)	Reference			
No	94 (71.8)	37 (28.2)	1.14 (0.76, 1.69)	0.528		
**Distance from the clinic**						
0-<5 km	993 (74.5)	339 (25.5)	Reference			
5-10 km	117 (73.6)	42 (26.4)	1.05(0.72, 1.53)		0.99 (0.67, 1.46)	0.960
>10 km	10 (45.5)	12 (54.5)	3.52 (1.51, 8.21)	0.004	3.30 (1.37, 7.97)	**0.008**
**Family history of hypertension**						
No	324 (76.6)	99 (23.4)	Reference			
Yes	810 (73.1)	298 (26.9)	1.20 (0.93, 1.56)	0.164	1.11 (0.84, 1.47)	0.463
**Experience of hypertensive emergency**						
No	1024 (73.8)	364 (26.2)	Reference			
Yes	110 (76.9)	33 (23.1)	0.84 (0.56, 1.27)	0.414		
**Experience of side effects of antihypertensive medications**						
No	1048 (74.9)	351 (25.1)	Reference			
Yes	86 (65.2)	46 (34.8)	1.60(1.09, 2.33)	0.015	1.63 (1.10, 2.43)	**0.016**
**Concern about the long-term side effects of medications**						
No	878 (77.6)	254 (22.4)	Reference			
Yes	256 (64.2)	143 (35.8)	1.93 (1.51, 2.47)	<0.001	1.72 (1.33, 2.24)	**<0.001**
**Usage of alternative medicine**						
No	1062 (75.3)	349 (24.7)	Reference			
Yes	72 (60.0)	48 (40.0)	2.03 (1.38, 2.98)	<0.001	1.93 (1.29, 2.88)	**0.001**
**Clinical factors**						
**Duration of hypertension**						
>10 years	276 (80.2)	68 (19.8)	Reference			
5-10 years	396 (75.1)	131 (24.9)	1.34 (0.97, 1.87)	0.081	0.27 (0.54, 0.18)	0.272
1-5 years	462 (70)	198 (30)	1.74 (1.27, 2.38)	<0.001	0.44 (0.12, 1.60)	0.211
**Duration of antihypertensive treatment**						
>10 years	267 (81.2)	62 (18.8)	Reference			
5-10 years	391 (75.2)	129 (24.8)	1.42 (1.01, 2.00)	0.043	2.34 (0.76, 7.13)	0.137
1-5 years	475 (69.8)	206 (30.2)	1.87 (1.36, 2.58)	<0.001	3.60 (0.96, 13.46)	0.057
**Frequency of taking medications**						
Once daily	1053 (73.9)	371 (26.1)	Reference			
Twice daily	67 (74.4)	23 (25.6)	0.97 (0.60, 1.59)	0.917		
Thrice daily	14 (82.4)	3 (17.6)	0.61 (0.17, 2.13)	0.437		
**Number of antihypertensive medications**						
1	593 (73.6)	213 (26.4)	Reference			
2	381 (75.6)	123 (24.4)	0.90 (0.70, 1.16)	0.415		
3	135 (73.4)	49 (26.6)	1.01 (0.70, 1.45)	0.955		
**Taking combined antihypertensive medications**						
Yes	46 (79.3)	12 (20.7)	Reference			
No	1088 (73.9)	385 (26.1)	1.36 (0.71, 2.59)	0.355		
**Total number of medications taken daily (all medications taken)**						
1-2	594 (72.9)	221 (27.1)	Reference			
3-4	421 (75.3)	138 (24.7)	0.88 (0.69, 1.13)	0.314		
>4	119 (75.8)	38 (24.2)	0.86 (0.58, 1.28)	0.450		
**Combination with antiplatelet medications**						
Yes	146 (73.4)	53 (26.6)	Reference			
No	988 (74.2)	344 (25.8)	0.96 (0.68, 1.34)	0.808		
**Combination with statins**						
Yes	761 (74.0)	268 (26.0)	Reference			
No	373 (74.3)	129 (25.7)	0.98 (0.77, 1.25)	0.884		
**Having other comorbidities**						
Yes	795 (74.4)	274 (25.6)	Reference			
No	339 (73.4)	123 (26.6)	1.05 (0.82, 1.35)	0.684		

Adhere is the reference. OR, odds ratio; aOR, adjusted odds ratio; Hosmer and Lemeshow test (P=0.888)

In the multivariate analysis, the number of antihypertensive medications taken was found to be the only factor associated with uncontrolled blood pressure. Taking three antihypertensive medications was positively associated with uncontrolled hypertension (aOR=1.46, 95% CI=1.05, 2.04) (Table 3).

**Table 3:** Factors associated with blood pressure control among the participants (n=1531).

Variable	Controlled blood pressure n(%)	Uncontrolled blood pressure n(%)	Univariate analysis		Multivariate analysis	
			OR (95% Cl)	P	aOR (95% Cl)	P
**Sociodemographic factors**						
**Age**						
≥60 years	428 (50.7)	416(49.3)	Reference			
<60 years	359 (52.3)	328 (47.7)	0.97 (0.77, 1.15)	0.547		
**Sex**						
Male	326 (48.8)	342 (51.2)	Reference			
Female	461 (53.4)	402 (46.6)	0.83 (0.68, 1.02)	0.073	0.87 (0.71, 1.08)	0.198
**Ethnicity**						
Chinese	292 (54.7)	242 (45.3)	Reference			
Malay	460 (49.8)	464 (50.2)	1.22 (0.98, 1.51)	0.072	1.15(0.92, 1.44)	0.232
Indian	20 (40.0)	30 (60.0)	1.81 (1.00,3.27)	0.049	1.77(0.97, 3.22)	0.061
Others	15 (65.2)	8 (34.8)	0.64 (0.27, 1.54)	0.323	0.68 (2.77, 1.66)	0.396
**Educational level**						
Tertiary	133 (50.8)	129 (49.2)	Reference			
Secondary	448 (51.8)	417(48.2)	0.96 (0.73, 1.27)	0.770		
Primary	206 (51.0)	198 (49.0)	0.99(0.73, 1.35)	0.954		
**Marital status**						
Single/divorced/widowed	141 (52.8)	126 (47.2)	Reference			
Married	646 (51.1)	618 (48.9)	1.07 (0.82, 1.40)	0.613		
**Occupation**						
Employed	318(50.1)	317(49.9)	Reference			
Unemployed	469 (52.3)	427 (47.7)	0.91 (0.75, 1.12)	0.382		
**Income**						
>RM 11,000	6 (37.5)	10(62.5)	Reference			
RM 5001-11,000	52 (47.3)	58 (52.7)	0.67 (0.23, 1.97)	0.466		
<RM 5000	728 (51.9)	676 (48.1)	0.56 (0.20, 1.54)	0.260		
**Patient-related factors**						
**Staying with family members**						
Yes	726 (51.6)	682 (48.4)	Reference			
No	61 (49.6)	62 (50.4)	1.08 (0.75, 1.56)	0.675		
**Receiving family support**						
Yes	721 (51.5)	679 (48.5)	Reference			
No	66 (50.4)	65 (49.6)	1.05 (0.73, 1.50)	0.807		
**Distance from the clinic**						
0-<5 km	683 (51.3)	649 (48.7)	Reference			
5-10 km	82 (51.6)	77 (48.4)	0.99 (0.71, 1.37)	0.944		
> 10 km	12(54.5)	10 (45.5)	0.88 (0.38, 2.04)	0.761		
**Family history of hypertension**						
No	214 (50.6)	209 (49.4)	Reference			
Yes	573 (51.7)	535 (48.3)	0.96 (0.76, 1.20)	0.694		
**Experience of hypertensive emergency**						
No	724 (52.2)	664 (47.8)	Reference			
Yes	63(44.1)	80 (55.9)	1.39 (0.98, 1.96)	0.066	1.24 (0.86, 1.79)	0.247
**Experience of side effects of antihypertensive medications**						
No	729 (52.1)	670 (47.9)	Reference			
Yes	58 (43.9)	74 (56.1)	1.39(0.97, 1.99)	0.074	1.33 (0.92, 1.92)	0.130
**Concern about the long-term side effects of medications**						
No	591 (52.2)	541 (47.8)	Reference			
Yes	196 (49.1)	203 (50.9)	1.13(0.90, 1.42)	0.89	1.72(1.33,2.24)	<0.001
**Usage of alternative medicine**						
No	733 (51.9)	678 (48.1)	Reference			
Yes	54 (45.0)	66 (55.0)	1.32 (0.91, 1.92)	0.145	1.40 (0.95,2.05)	0.088
**Clinical factors**						
**Duration of hypertension**						
>10 years	187 (54.4)	157 (45.6)	Reference			
5-10 years	264 (50.1)	263 (49.9)	1.19(0.90, 1.56)	0.218	1.55 (0.53,4.53)	0.428
1-5 years	336 (50.9)	324 (49.1)	1.15 (0.88, 1.49)	0.299	2.25 (0.62, 8.18)	0.218
**Duration of antihypertensive treatment**						
> 10 years	178 (54.1)	151 (45.9)	Reference			
5-10 years	260 (50.0)	260 (50.0)	1.18(0.89, 1.56)	0.244	0.83 (0.28, 2.46)	0.737
1-5 years	348 (51.1)	333 (48.9)	1.13(0.87, 1.47)	0.371	0.54 (0.15, 1.98)	0.353
**Frequency of taking medications**						
Once daily	736 (51.7)	688 (48.3)	Reference			
Twice daily	43 (47.8)	47 (52.2)	1.17(0.76, 1.79)	0.472		
Thrice daily	8(47.1)	9 (52.9)	1.20 (0.46, 3.14)	0.705		
**Number of antihypertensive medications**						
1	429 (53.2)	377 (46.8)	Reference			
2	262 (52.0)	242 (48.0)	1.05 (0.84, 1.31)	0.661	1.06 (0.85, 1.34)	0.600
3	81 (44.0)	103 (56.0)	1.45 (1.05,2.00)	0.025	1.46 (1.05, 2.04)	0.026
**Taking combined antihypertensive medications**						
Yes	35 (60.3)	23 (39.7)	Reference			
No	752 (51.1)	721 (48.9)	1.46 (0.85, 2.49)	0.167	1.63 (0.93, 2.85)	0.089
**Total number of medications taken daily (ail medications taken)**						
1-2	426 (52.3)	389 (47.7)	Reference			
3-4	281 (50.3)	278 (49.7)	1.08 (0.87, 1.34)	0.466		
>4	80 (51.0)	77 (49.0)	1.05 (0.75, 1.48)	0.763		
**Combination with antiplatelet medications**						
Yes	105 (52.8)	94 (47.2)	Reference			
No	682 (51.2)	650 (48.8)	0.94 (0.70, 1.27)	0.681		
**Combination with statins**						
Yes	523 (50.8)	506 (49.2)	Reference			
No	264 (52.6)	238 (47.4)	1.07 (0.87, 1.33)	0.517		
**Having other comorbidities**						
No	251 (54.3)	211 (45.7)	Reference			
Yes	536 (50.1)	533 (49.9)	1.18 (0.95, 1.47)	0.132	0.84 (0.67, 1.06)	0.137

Controlled blood pressure is the reference. OR, odds ratio; aOR, adjusted odds ratio; Hosmer and Lemeshow test (P=0.979)

Table 4 presents the association between adherence to antihypertensive medications and blood pressure control among the participants. The sociodemographic, patient-related and clinical factors were adjusted in the multivariate logistic regression model. After adjustment, the participants who reported being adherent to antihypertensive medications were found to be more likely to have well-controlled blood pressure (a0R=1.60, 95% CI=1.25, 2.05) (Table 4).

**Table 4:** Association between adherence to antihypertensive medications and blood pressure control among the participants (n=1531).

Variable	Controlled blood pressure n (%)	Uncontrolled blood pressure n (%)	Multivariate analysis^*^	
			aOR (95% CI)	P
**Medication adherence**				
Yes	615 (54.2)	519 (45.8)	1.60 (1.25, 2.05)	<0.001
No	172 (43.3)	225 (56.7)	Reference	

Controlled blood pressure is the reference. aOR, adjusted odds ratio; Hosmer and Lemeshow test (P=0.267).

* The model was adjusted for the sociodemographic, patient-related and clinical factors.

## Discussion

This study investigated the adherence to antihypertensive medications and blood pressure control among patients attending primary healthcare clinics in the Melaka Tengah District, Malaysia. Among the participants, 74.1% reported to have good adherence to antihypertensive medications. The adherence rate is higher in the present study than in a previously conducted study in Melaka Tengah in 1997 (44% for antihypertensive, antidiabetic and antiasthmatic drugs).^17^ Another study in primary healthcare revealed an adherence rate of 53.4% in 2012.^18^ The findings from these previous studies have shown an improvement of adherence to antihypertensive medications in Malaysia over the last two decades. This improvement could be attributed to health awareness programmes on medication adherence^19^ in Malaysia. In the UK, adherence was tested using liquid chromatography-tandem mass spectrometry among older adults with hypertension, and an adherence rate of 95.35% was reported.^20^ Therefore, adherence to antihypertensive medications could be further improved among patients in Malaysia, as it is related to blood pressure control.

In our study, 51.4% of the participants had blood pressure control at <140/90 mmHg. This rate is higher than that reported in the 2019 NHMS (45%).^4^ For further improvement of blood pressure control, multifaceted strategies including physical activity and lifestyle changes, dietary modification, smoking cessation, medication adherence and regular follow-up are recommended.^21^

The present study revealed that antihypertensive medication adherence differed among the ethnic groups. Similar findings were reported in a previous study wherein Chinese patients had better adherence to antihypertensive medications.^18^ On the contrary, ethnic differences were not associated with antihypertensive medication adherence in a study conducted in Selangor.^22^ A qualitative study demonstrated both positive and negative impacts of culture, social factors, beliefs, religiosity and spirituality on antihypertensive medication adherence in Malaysia.^23^ Therefore, there is a need for further exploration on culture, social factors and beliefs among ethnic groups to gain a better understanding of the influence of ethnic differences on adherence in the study setting.

Our findings contribute to the complex discourse on how education affects medication adherence, showing that the participants with secondary education were more likely to be non-adherent than those with tertiary education. Surprisingly, the adherence rate did not significantly differ between the participants with primary education and those with tertiary education. This finding contrasts with a report that indicated no significant association between education and adherence^24^ but aligns with another report within Malaysia that showed an association between better adherence and lower educational levels.^25^ Consistent with prior research, most of our participants received secondary education, while a smaller proportion had attained tertiary education. The varying findings between our study and previous studies highlight the need for deeper exploration into aspects such as health literacy of patients, beliefs of patients about medications and community support systems that facilitate adherence.

Similar to a previous systematic review,^26^ our study identified a significant increase in medication non-adherence among the patients residing at a considerable distance from the clinic but showed no significant difference for those within closer proximity. These discrepancies emphasise the complexity of healthcare access and its multifaceted influence on adherence, suggesting that factors beyond mere distance, such as transportation options and clinic wait times, may play critical roles in shaping patient behaviour.

The present findings highlight the importance of good communication with patients each time a new type of medicine is prescribed. In this study, the level of tolerance to antihypertension medications differed among our patients. Some did not experience any side effects. Among those who experienced side effects, mild symptoms such as giddiness and leg swelling to severe forms such as Stevens-Johnson syndrome and kidney damage were reported. Thus, it is of utmost importance for treating doctors to educate their patients regarding the benefits and possible side effects of each medication to ensure that patients maintain their faith and trust in the treatment. Notably, in our study, 8.6% of the patients who encountered side effects and 26.1% of the patients who were concerned about the long-term side effects of medications were more likely to be non-adherent, highlighting how immediate and anticipated adverse effects can deter patients from continuing their medication regimens.

Our patients who were taking traditional medicine were significantly more likely to be nonadherent to the prescribed medication regimens, echoing findings from Y:w et al. in Malaysia.^27^ Additionally, 11% of our patients who were concerned about the long-term consequences of medications opted for traditional medicine. This finding suggests that patients who are concerned about the side effects of western antihypertensive medications might be more inclined to seek alternative treatments, such as traditional medicine, which they may perceive as safer or more natural. Our finding underscores the global challenge of integrating traditional medicine with modern healthcare and highlights the importance of addressing these preferences in patient management to enhance medication adherence.

The current report emphasises the importance of considering the potential impact of prescribing multiple antihypertensive medications on patient adherence. One of these impacts is non-adherence to medications prescribed, consequently leading to uncontrolled blood pressure and increasing the risks of complications. Healthcare providers should be mindful of the potential challenges patients may face in adhering to complex medication regimens and work proactively to address these barriers. Strategies to improve adherence may include simplifying medication regimens, providing education and support to patients and utilising tools such as pill organisers and reminder systems.

Improving antihypertensive medication adherence is associated with achieving blood pressure control among patients. This significant association was observed in our study, highlighting the importance of addressing the challenges that hinder medication adherence. In the clinical setting, healthcare providers can help improve adherence by simplifying medication regimens^28^ and assigning tasks for nurse-led interventions that focus on increasing awareness of the importance of medication adherence.^29^ Counselling patients about the potential side effects of medications upon initiation of treatment, involving patients in the decision-making process and providing reassurance and support for managing side effects can also help improve medication adherence. In addition, using an innovative approach via mobile phones (mHealth) for self-management and medication reminders can benefit adherence to medications.^30^

### Strengths and limitations

One strength of our study is that the participants were recruited across primary healthcare clinics in the Melaka Tengah District, yielding a relatively large sample size. Further, the validated MyMAAT was utilised to measure adherence in the local setting. Another strength is the comprehensive assessment of the sociodemographic, patient-related and clinical factors of the patients, which helped in identifying the associated factors and gaining a better understanding of antihypertensive medication adherence.

This study also has some limitations. The participants were recruited from a single district, limiting the generalisability of the findings to a wider context in Malaysia. The participants’ ethnicity was not equally distributed across different groups, which might contribute to a sampling bias. Further, medication adherence was assessed as self-reported data, possibly yielding self-report bias and information bias. The MyMAAT used during data collection was previously validated among patients with diabetes. Therefore, the reliability of the findings among patients with hypertension might be limited. Another limitation is the cross-sectional study design, which was not able to capture changes in medication adherence and blood pressure control over time.

## Conclusion

The study population had good medication adherence and blood pressure control. Approximately 51.4% achieved blood pressure control, which is slightly higher than the proportion reported in the 2019 NHMS. Experiencing side effects, having concerns about long-term side effects and using alternative medicine were associated with poor adherence. Malay ethnicity, low literacy level and long distance from the clinic were associated with non-adherence. Since medication adherence was significantly associated with blood pressure control, patients with the associated factors of non-adherence should be closely monitored to improve the blood pressure control and prevent the adverse health outcomes.

### Recommendation

To improve adherence, healthcare providers could provide simplify the regimen (e.g. using combination pills and single daily doses); educate patients about medication adherence and hypertension, including the potential side effects of antihypertensive medications; engage patients in the decision-making process; provide re-assurance; and offer support for managing side effects during follow-up. Services for chronic diseases should be provided at community clinics, which are usually nearer patients’ residences.

The healthcare system could support and initiate innovative strategies to improve adherence such as using mHealth technology for self-management and providing reminders for medication and follow-up.

Further studies should explore the cultural factors and beliefs related to medication among different ethnic groups to gain a better understanding and offer better support to patients. The effectiveness of interventions in improving antihypertensive medication adherence should be further investigated.

## Data sharing statement

Data are available upon request to the authors.

**How does this paper make a difference in general practice?**

- The rate of antihypertensive medication adherence is 74.1% in the study population. There are factors that could help improve medication adherence and blood pressure control.
- Possible side effects of antihypertensive medications and concern about long-term side effects should be addressed by counselling and involving patients in decision-making to help improve adherence in a functioning comprehensive primary healthcare clinic.
- Healthcare providers could provide a simplified regimen (combination pills and single daily doses) to improve antihypertensive medication adherence.
- Ethnic variations in adherence patterns can help focus and refine future awareness and intervention strategies.
